# Prognostic implications of peritumoral vasculature in head and neck cancer

**DOI:** 10.1002/cam4.1910

**Published:** 2018-12-21

**Authors:** Michael Evans, Harry Michael Baddour Jr, Kelly R. Magliocca, Susan Müller, Sreenivas Nannapaneni, Amy Y. Chen, Sunjin Kim, Zhengjia Chen, Dong M. Shin, Andrew Y. Wang, Nabil F. Saba, Zhuo G. Chen

**Affiliations:** ^1^ Department of Hematology and Medical Oncology, Winship Cancer Institute Emory University School of Medicine Atlanta Georgia; ^2^ Department of Otolaryngology‐Head and Neck Surgery Emory University School of Medicine Atlanta Georgia; ^3^ Department of Pathology & Laboratory Medicine Emory University School of Medicine Atlanta Georgia; ^4^ Department of Biostatistics and Bioinformatics Emory Rollins School of Public Health Atlanta Georgia; ^5^ Ocean NanoTech LLC San Diego California

**Keywords:** blood vasculature, head and neck cancer, lymphatic vasculature, metastasis, prognosis

## Abstract

**Background:**

There is conflicting evidence regarding the role of peritumoral lymphatic vessel density (LVD) and blood microvessel density (MVD) in the metastasis and prognosis of head and neck squamous cell carcinoma (HNSCC). Existing studies are limited to one or two head and neck subsites and/or small sample sizes. A larger study incorporating multiple sub‐sites is needed to address the role of peritumoral LVD and MVD in HNSCC metastasis and prognosis.

**Methods:**

Tissue samples from 200 HNSCC cases were stained simultaneously using immunohistochemistry (IHC) for markers of peritumoral LVD (lymphatic vessel marker D240) and MVD (blood vessel marker CD31). Of the stained slides, 166 and 167 were evaluable for LVD and MVD, respectively. The results were then correlated with clinicopathologic features and patient outcomes.

**Results:**

Patients with metastatic disease were more likely to have high peritumoral MVD. Through multivariable analyses, MVD was not significantly related to DFS and OS, while low LVD was related to higher risk of disease progression and poor survival.

**Conclusions:**

Peritumoral MVD was found to be positively associated with metastasis, while LVD was found to be inversely related to both metastasis and progression of HNSCC. These findings may suggest a prognostic role of both peritumoral LVD and MVD in patients with HNSCC.

## 1 INTRODUCTION

Approximately 62 000 new cases of head and neck cancer (HNC) were expected in the United States in 2016 with nearly 13 000 expected cancer‐related deaths.^1^ Worldwide, HNC is responsible for almost 200 000 deaths each year and is the sixth most common cancer by incidence.^2^, ^3^ Squamous cell carcinoma of the head and neck (HNSCC) accounts for about 90% of cancers in this area. The subsites of the head and neck involved include the oral cavity, oropharynx, and larynx. Nearly, two‐thirds of patients present with locally advanced disease with a 5‐year overall survival rate (OS) of less than 50% secondary to regional recurrence, lymph node metastasis (LNM), and the development of second primary tumors (SPTs).^4^, ^5^

The propensity for tumor progression and metastasis is integrally associated with the peritumoral region known as the tumor microenvironment (TME).^6^, ^7^, ^8^ The TME consists of a variety of cells including endothelial, inflammatory, and immune cells as well as fibroblasts. In addition, the TME contains the extracellular matrix (ECM) and numerous signaling molecules including cytokines. Endothelial cells are responsible for the formation of vascular and lymphatic vessels, through the processes of angiogenesis and lymphangiogenesis, respectively. As with many epithelial tumors, HNSCC tends to metastasize via the lymphatic route more often than hematogenously; metastasis is thought to involve spread via existing vessels within the TME (ie, peritumoral) as well as invasion of new vessels formed within the primary tumor itself (ie, intratumoral).^6^, ^9^

The study of tumor biology and its correlation with clinical and pathologic variables allows for treatment teams to individualize therapy as well as improve patient education regarding treatment and prognosis. Currently, there is conflicting evidence regarding the role of lymphatic vessel density (LVD) and blood microvessel density (MVD) in HNSCC metastasis and prognosis.^10^, ^11^, ^12^ Most studies consist of small study populations and are limited to one or two head and neck sub‐sites. A larger study incorporating multiple sub‐sites is needed to address the role of LVD and MVD, particularly in the TME, on HNSCC metastasis and prognosis.

In our study, peritumoral lymphatic and blood vasculatures in 200 HNSCC tissue specimens were examined and correlations with patient outcomes were investigated in order to predict tumor behavior and guide treatment.

## 2 MATERIAL AND METHODS

### 2.1 Tissue samples and patient information

Using an Institutional Review Board‐approved consent for tissue acquisition, clinical samples for this study were obtained from surgical specimens from patients diagnosed with HNSCC from 1994 to 2003 at the Winship Cancer Institute of Emory University (Atlanta, GA). The primary treatment for these patients was surgery, and no prior treatment with radiation and/or chemotherapy was administered. Patient samples consisted of primary SCC samples from 99 patients with LNM and 101 patients without LNM upon presentation. None of the patients developed metastases within 2 years of the initial procedure. The clinical information associated with the samples was obtained from the surgical pathology reports in the Department of Pathology at Emory University according to the regulations of the Health Insurance Portability and Accountability Act. The clinicopathologic parameters characterized, including age, sex, and disease stage, are listed in Table 1. Each patient's overall survival (OS) and disease‐free survival (DFS) were documented through June 2012.

**Table 1:** Patient characteristics by metastasis status

Covariate	Level	All patients (N = 200)	Metastasis status		*P*‐value*
			Met (N = 101)	Non‐Met (N = 99)	
Age at diagnosis	Median (Range)	61 (22‐93)	59 (23‐93)	63 (22‐89)	0.163
Sex	Female	70 (35)	33 (47.14)	37 (52.86)	0.486
	Male	130 (65)	68 (52.31)	62 (47.69)	
Grade	WD	32 (16)	3 (9.38)	29 (90.63)	**<0.001**
	MD	132 (66)	75 (56.82)	57 (43.18)	
	PD	26 (13)	13 (50)	13 (50)	
	NK	10 (5)	10 (100)	0 (0)	
Stage	I	41 (20.5)	0 (0)	41 (100)	**<0.001**
	II	31 (15.5)	0 (0)	31 (100)	
	III	29 (14.5)	17 (58.62)	12 (41.38)	
	IV	99 (49.5)	84 (84.85)	15 (15.15)	
T Stage	T1	65 (32.5)	24 (36.92)	41 (63.08)	0.062
	T2	69 (34.5)	38 (55.07)	31 (44.93)	
	T3	29 (14.5)	17 (58.62)	12 (41.38)	
	T4	37 (18.5)	22 (59.46)	15 (40.54)	
N Stage	N0	99 (49.5)	0 (0)	99 (100)	NA
	N1	19 (9.5)	19 (100)	0 (0)	
	N2	74 (37)	74 (100)	0 (0)	
	N3	8 (4)	8 (100)	0 (0)	
N Stage:binary	N0	99 (49.5)	0 (0)	99 (100)	NA
	N1‐N3	101 (50.5)	101 (100)	0 (0)	
Site	L	61 (30.5)	28 (45.9)	33 (54.1)	**<0.001**
	OC	101 (50.5)	40 (39.6)	61 (60.4)	
	OP	38 (19)	33 (86.84)	5 (13.16)	

Covariate	Level	All patients (N = 167)	Metastasis status		*P*‐value*
			Met (N = 90)	Non‐Met (N = 77)	
MVD:cut by ROC (High ≥ 40.667)	High	65 (38.92)	55 (84.62)	10 (15.38)	**<0.001**
	Low	102 (61.08)	35 (34.31)	67 (65.69)	
MVD:cut by DFS optimal (High ≥ 53)	High	26 (15.57)	21 (80.77)	5 (19.23)	**0.003**
	Low	141 (84.43)	69 (48.94)	72 (51.06)	
MVD:cut by OS optimal (High ≥ 39.667)	High	67 (40.12)	56 (83.58)	11 (16.42)	**<0.001**
	Low	100 (59.88)	34 (34)	66 (66)	

Covariate	Level	All patients (N = 166)	Metastasis status		*P*‐value*
			Met (N = 90)	Non‐Met (N = 77)	
LVD:cut by ROC (High ≥ 3.001)	High	132 (79.52)	64 (48.48)	68 (51.52)	**0.003**
	Low	34 (20.48)	26 (76.47)	8 (23.53)	
LVD:cut by DFS optimal (High ≥ 8.667)	High	70 (42.17)	36 (51.43)	34 (48.57)	0.538
	Low	96 (57.83)	54 (56.25)	42 (43.75)	
LVD:cut by OS optimal (High ≥ 2.667)	High	137 (82.53)	68 (49.64)	69 (50.36)	**0.010**
	Low	29 (17.47)	22 (75.86)	7 (24.14)	

Covariate		Median (Range)	Metastasis status		*P*‐value*
			Met	Non‐Met	
MVD		36 (11.67‐108)	43.67 (16.33‐108)	29.67 (11.67‐60)	**<0.001**
LVD		7.42 (0‐51.67)	7 (0‐51.67)	7.83 (0‐32.33)	0.154

Data are presented as number of patients (%) or median (range).

* The *P*‐value is calculated by Wilcoxon rank‐sum test for numerical covariates; and chi‐squared test or Fisher's exact test for categorical covariates, where appropriate.

Significant *P*‐value is bolded.

### 2.2 Immunohistochemical (IHC) analysis

Immunohistochemical analyses on the 200 formalin‐fixed paraffin‐embedded (FFPE), human specimens were initially performed according to the following protocol. In brief, after deparaffinization with xylene and rehydration with ethanol, endogenous peroxidase activity was blocked by incubating the slides in 3% hydrogen peroxide with methanol for 15 minutes. To retrieve the antigens, the tissue slides were heated in a microwave oven in 100 mmol/L of sodium citrate buffer (pH 6.0) for 10 minutes and then allowed to remain at room temperature for 20 minutes. After being washed in PBS, the slides were incubated with 2.5% normal horse serum (Vector Laboratories, Burlingame, CA) to decrease the background signal. Next, the slides were incubated with two primary antibodies simultaneously (CD31: ab76533, rabbit monoclonal, 1:200 dilution and D240: ab77854, mouse monoclonal, 1:80 dilution, Abcam Inc Cambridge, MA) overnight at 4°C, left at room temperature for 20 minutes, and washed with PBS. The slides were then incubated with secondary antibody for 20 minutes at room temperature and with DAKO EnVision™ G2 Doublestain system (Rabbit/Mouse: DAB/Permanent Red) following the manufacturer's instructions (DAKO North America, Inc Carpinteria, CA).

### 2.3 MVD and LVD analyses

**Figure 1:**
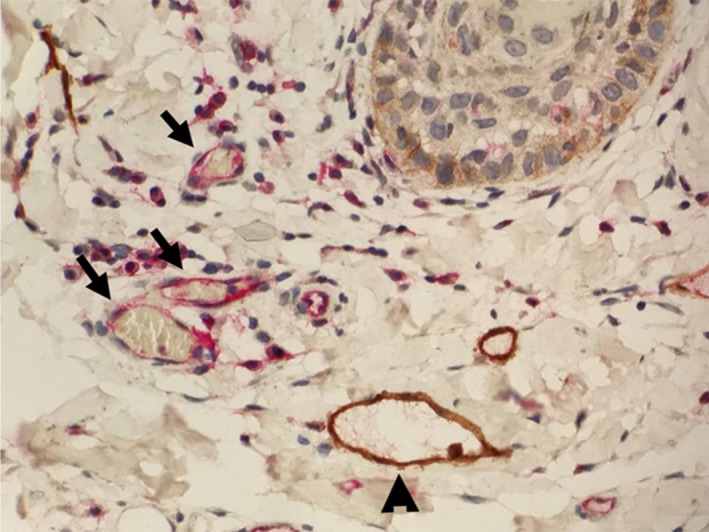
MVD and LVD staining in peritumoral region of HNSCC. Anti‐CD31 antibody (Rabbit, Abcam Inc) targets a transmembrane glycoprotein selectively expressed on hematopoietic progenitor cells and stains brown, depicting MVD indicated by ▲. Anti‐D240 antibody (Mouse, Abcam Inc) reacts with an O‐linked sialoglycoprotein found on lymphatic endothelium and stains red, depicting LVD indicated by 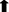 (400× Magnification)

Two investigators (HMB and KRM) analyzed and quantified peritumoral MVD and LVD for each specimen. Peritumoral was defined as <500 microns of the tumor border but not contained within the tumor itself (intratumoral). Each investigator was blinded to patients’ outcomes. Slides stained for CD31 and D240 were counted to determine MVD and LVD, respectively. A representative image of CD31 and D240 staining is shown in Figure 1. Each slide was initially examined by light microscopy at ×100 magnification using a Chalkley grid. At this magnification, the three areas with the highest number of stained vessels were identified as “hot spots”.^13^ Vessels in each of these “hot spots” were then counted using x400 magnification. Identification of vessels was performed using the method specified by Weidner, in which “any brown staining endothelial cell or cell cluster that was clearly separate from adjacent microvessels, tumor cells and other connective tissue elements were considered a single, countable microvessel”.^14^, ^15^, ^16^ With this method, MVD and LVD are expressed as the number of stained vessels per optical field. No counts were performed in areas of necrosis or inflammation. Sections in which three “hot spots” could not be identified were excluded from further analysis. If the two investigators scored differences of greater than ten vessels per high‐power optical field, sections were reviewed again until a consensus was reached. Once this happened, the vessel counts in each of the three “hot spots” were averaged to yield an average MVD and LVD.

### 2.4 Statistical analysis

Clinical characteristics were compared between patients with and without metastasis using the Wilcoxon rank‐sum test for numerical covariates and chi‐squared or Fisher's exact test for categorical covariates, where appropriate. Univariate association of MVD or LVD with patient characteristics was examined with the Kruskal‐Wallis test and Spearman's rank correlation coefficient.

To estimate the ability of a single biomarker or multiple biomarkers to predict metastasis status, a logistic regression model was used. Multivariate analysis of metastasis was conducted by entering variables into a logistic regression model and using a backward variable selection method with an alpha level of removal of 0.1. To further estimate the ability of a single biomarker to predict metastasis status, receiver operating characteristic (ROC) curves were created with an area under the curve (AUC) measured. The cut‐off values to obtain 90% sensitivity and 90% specificity were estimated. To obtain the optimal cut‐off points with the best discrimination power for metastasis status, sensitivity and specificity pairs were obtained in the logistic regression under all the possible thresholds. The optimal cut‐off point of each single biomarker and the combined biomarker was calculated where the maximum sum of sensitivity and specificity was achieved.

Survival functions were estimated by the Kaplan‐Meier method and a log‐rank test was used to assess the difference in DFS or OS between patients with high or low biomarker levels.^17^ A Cox proportional hazards model was employed to examine the effect of protein expression levels and covariates on DFS and OS.^18^ The proportional hazards assumption was also checked. Multivariable analysis was conducted by entering variables into a Cox proportional hazards model and using a backwards variable selection method with an alpha removal of 0.1. All analyses were performed using SAS 9.3 (SAS Institute, Inc, Cary, NC) with a significance level of 0.05.

## 3 RESULTS

### 3.1 Association of patients’ characteristics and MVD and LVD with LNM by univariate analyses

As shown in Table 1, initial analysis of 200 patients’ tissue samples consistently showed that disease stage, grade of differentiation, and tumor site were significantly associated with LNM while T‐stage was only moderately associated with LNM. HNSCC in the oropharynx (OP) had a higher rate of LNM than that in the oral cavity (OC) and larynx (L; *P* < 0.001).

Among 200 cases, MVD was quantifiable in 167 tissue samples and LVD was quantifiable in 166 slides. Univariate analysis showed that peritumoral MVD was significantly associated with LNM (*P* < 0.001), while no significant association was observed between peritumoral LVD and LNM (*P* = 0.154; Table 1).

Further analyses showed that high MVD was significantly associated with higher disease stage (*P* < 0.001) and N‐stage (*P* < 0.001), while low LVD was significantly associated with high disease stage (*P* = 0.015) and T‐stage (*P* = 0.001; Table S1). Furthermore, HNSCC in both OP and OC showed higher LVD than that in L (*P* = 0.021).

### 3.2 Multivariate association and prediction of metastasis status with MVD and LVD and other covariates

In a multivariable model, high MVD (*P* < 0.001), low LVD (*P* = 0.002), and OP disease (*P* = 0.013) were significantly associated with LNM (Table 2).

**Table 2:** Multivariable model of metastasis after adjusting for 2 biomarkers and covariates

Covariate	Level	Metastasis status = Met		
		Odds ratio (95% CI)	OR *P*‐value	Type3 *P*‐value
MVD		1.12 (1.07‐1.17)	**<0.001**	**<0.001**
LVD		0.91 (0.86‐0.97)	**0.002**	**0.002**
Site	OP	5.56 (1.43‐21.62)	**0.013**	**0.013**
	L	0.67 (0.26‐1.76)	0.416	
	OC	—	—	

Of 200, 162 observations were used in the multivariable logistic model.

Backward selection with an alpha level of removal of 0.1 was used. The following variables were removed from the model: Age at diagnosis, Chemotherapy, Sex, Smoking, T Stage, Diagnosis Year, and Grade.

Hosmer and Lemeshow Goodness‐of‐Fit Test statistic=4.154; *P*‐value = 0.84 (fitted model was an adequate model).

Likelihood Ratio test statistic =88.47; *P*‐value <0.001 (The overall logistic regression model was significant).

AUC (area under the Receiver Operating Characteristic [ROC] curve) = 0.8872; *P*‐value <0.001.

Significant *P*‐value is bolded.

**Figure 2:**
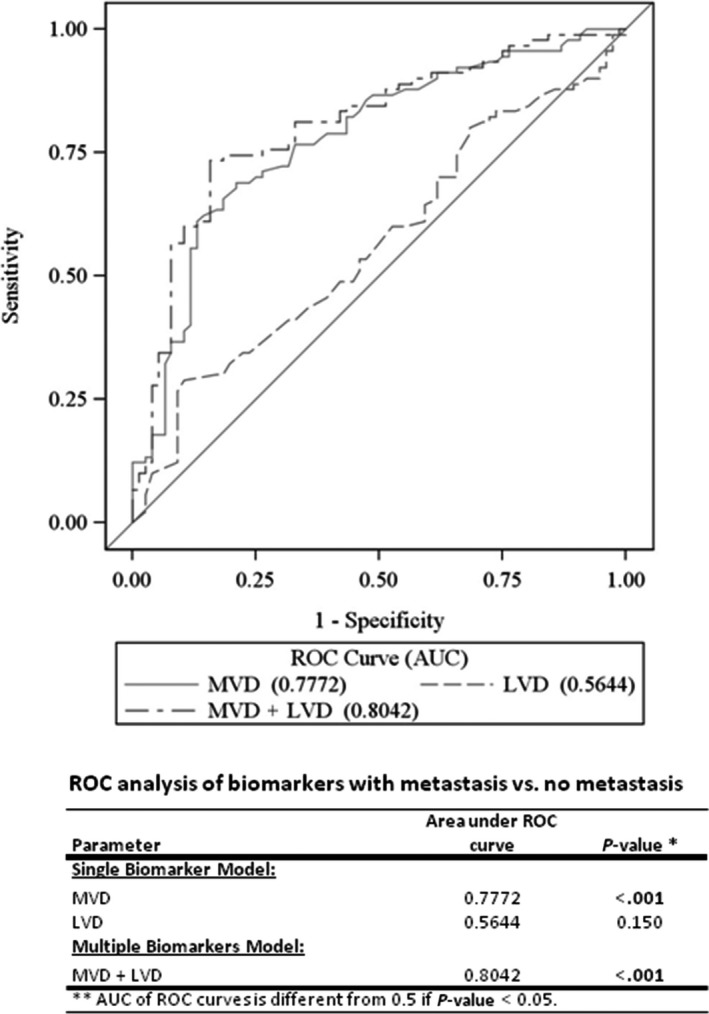
ROC curves of the studied biomarkers. ROC analysis shows MVD, LVD, MVD + LVD and their respective area under the curve (AUC) for predicting patient's metastasis status

We performed ROC analyses to compare the power of LNM prediction using MVD and LVD, both alone and in combination. As shown in Figure 2, the combination of MVD and LVD had a stronger predictive discrimination power (AUC: 0.8042) than either MVD or LVD alone. In this model, the maximized sum of sensitivity and specificity was 85.1% and 77.3%, respectively, for LNM prediction (Table S2).

### 3.3 Univariate survival analysis of DFS and OS

**Figure 3:**
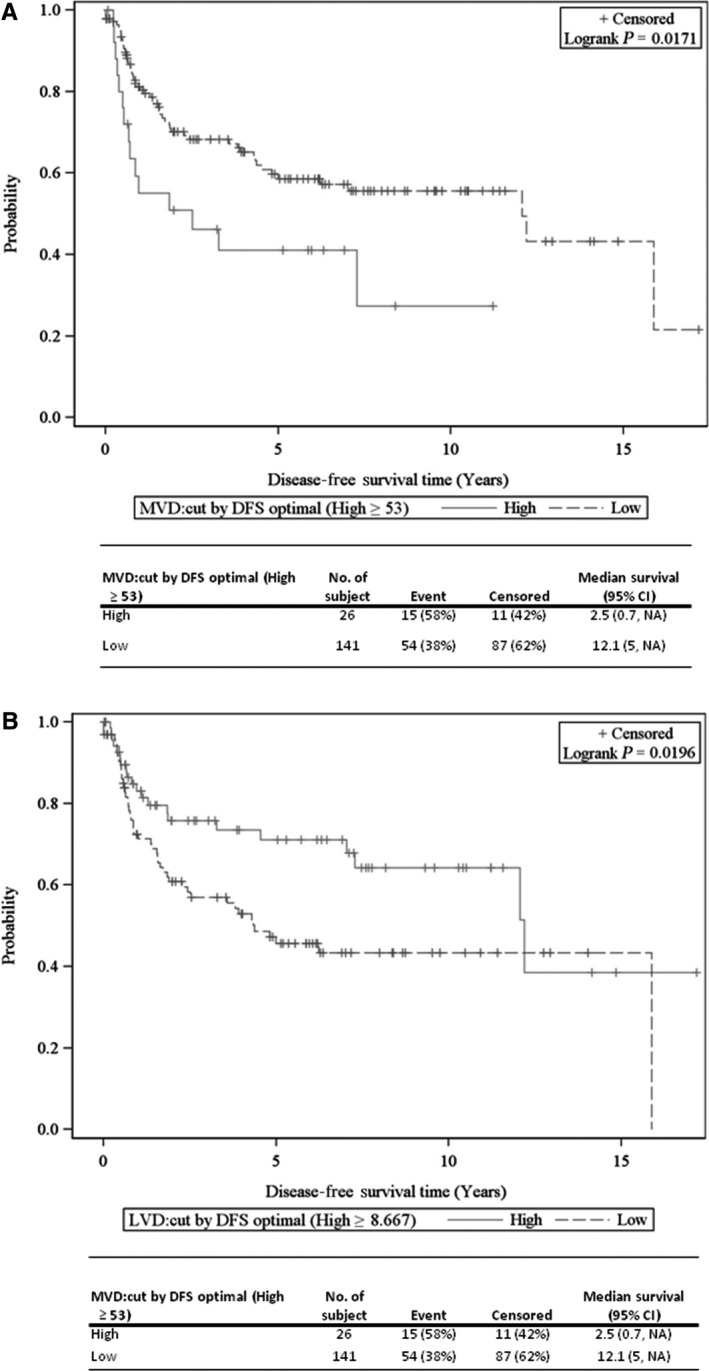
KM curves of disease‐free survival (DFS) for the studied biomarkers. A, MVD effect on DFS determined by an optimal cut‐point driven by DFS analysis (High ≥ 53). B, LVD effect on DFS determined by an optimal cut‐point driven by DFS analysis (High ≥ 8.667).

Patients with LNM had a higher risk of disease progression and poorer overall survival than those without LNM (*P = *0.043 and <0.001, respectively; Tables S3 and S4). A low MVD and high LVD were significantly associated with DFS after being dichotomized by the optimal cut‐off point driven by survival analysis (*P* = 0.017 and 0.020, respectively, Figures 3A,B, and Table S3). Similarly, low LVD showed a highly significant correlation with OS after being dichotomized by the optimal cut‐off point driven by survival analysis (*P* < 0.001; Table S4).

### 3.4 Multivariable survival analysis of DFS and OS with MVD and LVD and covariates

In a multivariable model, high LVD was significantly associated with DFS (*P* = 0.022; Table 3). Furthermore, high LVD (*P* = 0.001), lack of LNM (*P* = 0.032), female sex (*P* = 0.002), and OP disease (*P* < 0.001) were all significantly associated with longer OS (Table 3).

**Table 3:** Multivariable DFS and OS analysis with MVD and LVD

(A) Covariate	Level	Disease‐free survival time (years)		
		Hazard ratio	HR *P*‐value	Type3 *P*‐value
MVD:cut by DFS optimal (High ≥ 53)	High	1.62 (0.89‐2.96)	0.117	0.117
	Low	—	—	
LVD:cut by DFS optimal (High ≥ 8.667)	High	0.54 (0.32‐0.91)	**0.022**	**0.022**
	Low	—	—	
Site	OP	0.45 (0.21‐0.94)	**0.033**	0.091
	L	0.73 (0.41‐1.31)	0.296	
	OC	—	—	
Age at diagnosis		1.03 (1.00‐1.05)	**0.037**	**0.037**

(B) Covariate	Level	Overall survival time (years)		
		Hazard ratio	HR *P*‐value	Type3 *P*‐value
MVD:cut by OS optimal (High ≥ 39.667)	High	1.13 (0.70‐1.83)	0.610	0.610
	Low	—	—	
LVD:cut by OS optimal (High ≥ 2.667)	High	0.43 (0.26‐0.72)	**0.001**	**0.001**
	Low	—	—	
Metastasis status	Met	1.78 (1.05‐3.02)	**0.032**	**0.032**
	Non‐Met	—	—	
Sex	Male	2.08 (1.31‐3.33)	**0.002**	**0.002**
	Female	—	—	
Site	OP	0.32 (0.17‐0.62)	**<0.001**	**0.003**
	L	0.78 (0.48‐1.27)	0.318	
	OC	—	—	
Age at diagnosis		1.04 (1.02‐1.06)	**<0.001**	**<0.001**

Of 200, 164 observations were used in the multivariable Cox proportional hazards model.

Backward selection with an alpha level of removal of 0.1 was used. The following variables were removed from the model: (A) Metastasis status, Radiation, Sex, Smoking, T Stage, and Grade; (B) Radiation, Smoking, T Stage, Diagnosis Year, and Grade.

MVD and LVD were forced in the model.

Significant *P*‐value is bolded.

## 4 DISCUSSION

A large body of evidence links blood vessel angiogenesis with metastasis.^9^, ^19^, ^20^, ^21^ It has been demonstrated that high MVD is associated with both metastasis and poor prognosis in patients with head and neck cancers.^9^, ^22^ Lymphatic vasculature has also been shown to play a role, with high LVD being associated with poor prognosis in gastric, non‐small‐cell lung, and head and neck cancers.^10^, ^11^, ^23^, ^24^, ^25^, ^26^, ^27^, ^28^ Many of these studies have distinguished between peritumoral and intratumoral vessel densities. In HNSCC, high intratumoral LVD has been reported to be associated with higher risk of local recurrence,^10^ cervical nodal metastases,^11^, ^29^ and worse overall survival.^12^ Kyzas et al^30^ reported that high intratumoral LVD was associated with worse overall survival in HNSCC while peritumoral LVD had no influence on outcome. Similarly, Frech et al^29^ showed that high intratumoral LVD was associated with cervical nodal metastasis, but could not demonstrate an effect of peritumoral LVD. Our observation suggests that the biological function between intratumoral and peritumoral LVD may be different.

The mechanisms by which HNSCC can metastasize via lymphatic vessels have yet to be fully elucidated.^30^ Furthermore, the findings of the aforementioned studies suggest that intratumoral and peritumoral lymphatic vessels differ in their effects on disease progression. Padera et al^31^ postulated that intratumoral lymphatics are essentially nonfunctional, while lymphatics in the tumor microenvironment are the sites for lymphatic metastasis. However, the present study does not support this theory. Our data demonstrate that peritumoral LVD is inversely related to disease progression and directly related to disease‐free survival and overall survival. Additionally, we demonstrate that peritumoral MVD is inversely related to disease‐free survival. Our simultaneous investigation of peritumoral MVD and LVD is a novel strength of our study, as is our analysis of tumor biopsies taken prior to any treatment. Lymphatic vessels, as part of the immune system, play a role in the regulation of tumor immunity in the TME.^32^, ^33^ A recent publication has suggested that lymphatic vessels not merely conduits for fluid and immune cell transport. Accumulated data in the past several years indicate that lymphatic endothelial cells support T‐cell survival, inhibit exaggerated T‐cell proliferation during immune response, and maintain T‐cell memory.^34^ Whether peritumoral LVD is correlated with T‐cell and B‐cell populations and sensitivity to immune therapy deserves further investigation.

Our study has some limitations. While we analyzed both MVD and LVD in the peritumoral region, we did not investigate intratumoral vasculature. We also encountered some technical difficulties with staining certain samples, mainly cartilage and salivary tissue. Due to the nature of retrospective samples from a pre‐existing sample set, variability in the amount of tumor present on each slide was encountered. In cases with little residual tumor on the slide limited selection of sites at which one can score peritumoral vessels. By studying a spectrum of primary site HNSCC the variability of tissue types surrounding the tumor or invaded by tumor increased. A sample of carcinoma infiltrating regional lamina propria, skeletal muscle, minor salivary gland, tonsillar lymphoid tissue and/or abutting laryngeal cartilages would likely have a different ratio of lymphatics/blood vessel density depending on the environment. Finally, we do not have information regarding HPV status for the patients with OP disease because the tissues samples were collected before 2003. Therefore, our study did not consider HPV as a factor in the statistical analysis.

In conclusion, our study focused on the TME and revealed that high peritumoral MVD is positively related to metastasis, while high peritumoral LVD has a negative relationship with both metastasis and progression of HNSCC. Our findings stress the importance of distinguishing between peritumoral and intratumoral histologic analysis, and suggest a potential prognostic utility for sampling and assessing the tumor microenvironment in head and neck cancers. Future investigations could compare peritumoral and intratumoral MVD and LVD within the same patients and assess their correlations with metastasis and overall survival.

## CONFLICTS OF INTEREST

No potential conflicts of interest to disclose.
