# Supplementary material for: Prognostic implications of peritumoral vasculature in head and neck cancer
Source: Cancer Med. 2018 Dec 21;8(1):147–54. doi: 10.1002/cam4.1910 (PMC6346230; doi:10.1002/cam4.1910)
Supplement: Supplementary file 1 [file CAM4-8-147-s001.docx]

**Table S1: Univariate association of MVD and LVD with covariates**

|  | | | **MVD** | |  |
| --- | --- | --- | --- | --- | --- |
|  | | | **______________________________** | |  |
| **Variable** | **Level** | **N** | **Median (Range)** | **Kruskal-Wallis P-value** | |
| Sex | Male | 107 | 37 (11.67 - 80) | 0.967 | |
|  | Female | 60 | 35 (12 - 108) |  |  |
|  |  |  |  |  | |
| Grade | WD | 19 | 28.67 (15.33 - 60) | 0.201 | |
|  | MD | 117 | 38 (11.67 - 80) |  |  |
|  | PD | 22 | 34 (14.33 - 108) |  |  |
|  | NK | 9 | 42 (32.33 - 52.33) |  |  |
|  |  |  |  |  |  |
| Stage | I | 23 | 29.33 (17.33 - 60) | **<.001** | |
|  | II | 30 | 33.33 (11.67 - 57.33) |  |  |
|  | III | 25 | 36.67 (15.33 - 108) |  |  |
|  | IV | 89 | 40.67 (12 - 80) |  |  |
|  |  |  |  |  | |
| T Stage | T1 | 42 | 36.17 (17.33 - 108) | 0.828 | |
|  | T2 | 64 | 35.17 (11.67 - 80) |  |  |
|  | T3 | 27 | 36.67 (15.33 - 65.33) |  |  |
|  | T4 | 34 | 36.5 (12 - 71) |  |  |
|  |  |  |  |  | |
| N Stage | N0 | 77 | 29.67 (11.67 - 60) | **<.001** | |
|  | N1 | 18 | 43.83 (17 - 71) |  |  |
|  | N2 | 64 | 44.5 (16.33 - 108) |  |  |
|  | N3 | 8 | 36.67 (27.67 - 53) |  |  |
|  |  |  |  |  | |
| N Stage:binary | N0 | 77 | 29.67 (11.67 - 60) | **<.001** | |
|  | N1-N3 | 90 | 43.67 (16.33 - 108) |  |  |
|  |  |  |  |  | |
| Site | OP | 32 | 40.67 (17 - 69) | 0.317 | |
|  | L | 55 | 34.67 (14.33 - 64.33) |  |  |
|  | OC | 80 | 35.33 (11.67 - 108) |  |  |
| Age at diagnosis |  | 167 | **Spearman CC**  0.000 | **Spearman P-val**  0.998 | |

|  | | | **LVD** | |  |
| --- | --- | --- | --- | --- | --- |
|  | | | **______________________________** | |  |
| **Variable** | **Level** | **N** | **Median (Range)** | **Kruskal-Wallis P-value** |  |
| Sex | Male | 106 | 8.67 (0 - 51.67) | **0.016** |  |
|  | Female | 60 | 5.83 (0 - 32.33) |  | |
|  |  |  |  |  | |
| Grade | WD | 19 | 7.5 (1.67 - 32.33) | 0.230 |  |
|  | MD | 116 | 7.83 (0 - 51.67) |  |  |
|  | PD | 22 | 5 (0.33 - 21) |  |  |
|  | NK | 9 | 9 (0 - 28) |  |  |
|  |  |  |  |  |  |
| Stage | I | 22 | 12 (3.5 - 32.33) | **0.015** |  |
|  | II | 30 | 7.58 (1.33 - 21) |  |  |
|  | III | 25 | 6.67 (0 - 21.33) |  |  |
|  | IV | 89 | 6.33 (0 - 51.67) |  |  |
|  |  |  |  |  |  |
| T Stage | T1 | 41 | 9 (2 - 32.33) | **0.001** |  |
|  | T2 | 64 | 7.67 (0.33 - 28) |  |  |
|  | T3 | 27 | 6.67 (0 - 16.67) |  |  |
|  | T4 | 34 | 4.33 (0 - 51.67) |  |  |
|  |  |  |  |  |  |
| N Stage | N0 | 76 | 7.83 (0 - 32.33) | 0.406 |  |
|  | N1 | 18 | 7.17 (1.33 - 28) |  |  |
|  | N2 | 64 | 7.17 (0 - 51.67) |  |  |
|  | N3 | 8 | 5.17 (0.33 - 20.33) |  |  |
|  |  |  |  |  |  |
| N Stage:binary | N0 | 76 | 7.83 (0 - 32.33) | 0.153 |  |
|  | N1-N3 | 90 | 7 (0 - 51.67) |  |  |
|  |  |  |  |  |  |
| Site | OP | 32 | 8.83 (0.33 - 51.67) | **0.021** |  |
|  | L | 55 | 5 (0 - 32.33) |  |  |
|  | OC | 79 | 8 (0 - 26) |  |  |
|  |  |  |  |  |  |
| Smoking | Yes | 135 | 8 (0 - 51.67) | 0.175 |  |
|  | No | 20 | 6.5 (0.67 - 18.67) |  |  |
| Age at diagnosis |  | 167 | **Spearman CC**  0.000 | **Spearman P-val**  0.998 |  |
|  | | | | |  |

**Table S2: Summary of cut-off points for different sensitivities and specificities with metastasis vs. no metastasis**

| **Parameter** | **Sensitivity = 90%** | **Specificity = 90%** | **Maximize sum of specificity and sensitivity** | **≥ 50% to be Metastasis** |
| --- | --- | --- | --- | --- |
| **Single Biomarker Model:** | | | | |
| MVD | Specificity=53.2%  MVD=30.333 | Sensitivity=62.2%  MVD = 39.667 | Sensitivity=61.1%  Specificity=87.0%  MVD =40.667 | MVD ≥ 35.333 |
|  |  |  |  |  |
| LVD | Specificity=18.4%  LVD =15.671 | Sensitivity=28.9%  LVD =3.001 | Sensitivity=28.9%  Specificity=89.5%  LVD =3.001 | LVD ≤ 19.005 |
| **Unadjusted Multiple Biomarkers Model:** | | | | |
| Cut-off = 0.0978*MVD-0.0699*LVD | Specificity=48.7%  Cut-off=2.249 | Sensitivity=61.1%  Cut-off= 3.363 | Sensitivity=73.3%  Specificity=84.2%  Cut-off=3.117 | Cut-off ≥ 2.814 |
|  |  |  |  |  |
| **Covariate-adjusted Multiple Biomarkers Model:** | | | | |
| Cut-off = 0.1146*MVD-0.0929*LVD+1.7162*Site_OP-0.4007*Site_L+2.1883*Radiation * | Specificity=77.3%  Cut-off=4.916 | Sensitivity=74.7%  Cut-off= 5.475 | Sensitivity=85.1%  Specificity=77.3%  Cut-off=4.915 | Cut-off ≥ 4.98 |
|  |  |  |  |  |
| * Site_OP=1 if site=OP and 0 o.w; Site_L=1 if site=L and 0 o.w.; and Radiation=1 if radiation and 0 o.w. | | | | |

**Table S3: Univariate DFS analysis**

|  | | | **Disease Free Survival Time (Years)** | | | |
| --- | --- | --- | --- | --- | --- | --- |
|  | | | **----------------------------------------** | | | |
| **Covariate** | **Level** | **N** | **Hazard Ratio (95% CI)** | **HR P-value** | **Log-rank P-value** | |
| Metastasis status | Met | 101 | 1.58 (1.01-2.49) | **0.045** | **0.043** | |
|  | Non-Met | 99 | - | **-** |  |  |
|  | | | | | | |
| Sex | Male | 130 | 1.01 (0.64-1.60) | 0.968 | 0.968 | |
|  | Female | 70 | - | **-** |  |  |
|  | | | | | | |
| Grade | WD | 32 | 1.22 (0.34-4.32) | 0.759 | 0.606 | |
|  | MD | 132 | 1.66 (0.52-5.31) | 0.397 |  |  |
|  | PD | 26 | 1.84 (0.52-6.53) | 0.346 |  |  |
|  | NK | 10 | - | **-** |  |  |
|  | | | | | | |
| Stage | I | 41 | 0.70 (0.40-1.23) | 0.218 | 0.086 | |
|  | II | 31 | 0.73 (0.38-1.38) | 0.331 |  |  |
|  | III | 29 | 0.38 (0.17-0.85) | **0.019** |  |  |
|  | IV | 99 | - | **-** |  |  |
|  | | | | | | |
| T Stage | T1 | 65 | 0.66 (0.35-1.22) | 0.186 | 0.564 | |
|  | T2 | 69 | 0.73 (0.40-1.35) | 0.313 |  |  |
|  | T3 | 29 | 0.65 (0.30-1.43) | 0.289 |  |  |
|  | T4 | 37 | - | **-** |  |  |
|  | | | | | | |
| N Stage | N0 | 99 | 0.99 (0.24-4.11) | 0.985 | **0.043** | |
|  | N1 | 19 | 2.61 (0.57-11.96) | 0.218 |  |  |
|  | N2 | 74 | 1.43 (0.34-6.00) | 0.624 |  |  |
|  | N3 | 8 | - | **-** |  |  |
|  | | | | | | |
| N Stage:binary | N0 | 99 | 0.63 (0.40-0.99) | **0.045** | **0.043** | |
|  | N1-N3 | 101 | - | **-** |  |  |
|  | | | | | | |
| Site | OP | 38 | 0.46 (0.24-0.90) | **0.022** | **0.043** | |
|  | L | 61 | 0.68 (0.41-1.14) | 0.147 |  |  |
|  | OC | 101 | - | **-** |  |  |
|  | | | | | | |
| Smoking | Yes | 159 | 0.87 (0.48-1.58) | 0.639 | 0.639 | |
|  | No | 29 | - | **-** |  |  |
|  | | | | | | |
| MVD:cut by ROC (High >= 40.667) | High | 65 | 1.36 (0.83-2.22) | 0.223 | 0.222 | |
|  | Low | 102 | - | **-** |  |  |
|  | | | | | | |
| MVD:cut by DFS optimal (High >= 53) | High | 26 | 1.99 (1.12-3.54) | **0.019** | **0.017** | |
|  | Low | 141 | - | **-** |  |  |
|  | | | | | | |
| LVD:cut by ROC (High >= 3.001) | High | 132 | 0.65 (0.37-1.15) | 0.140 | 0.137 | |
|  | Low | 34 | - | **-** |  |  |
|  | | | | | | |
| LVD:cut by DFS optimal (High >= 8.667) | High | 70 | 0.55 (0.33-0.91) | **0.021** | **0.020** | |
|  | Low | 96 | - | **-** |  |  |
|  | | | | | | |
| 0.0978*MVD-0.0699*LVD:cut by DFS optimal (High >= 3.1247) | High | 75 | 1.75 (1.08-2.84) | **0.024** | **0.022** |  |
|  | Low | 91 | - | **-** |  |  |
|  | | | | | |  |
| MVD |  | 167 | 1.01 (0.99-1.03) | 0.196 | **-** |  |
|  | | | | | |  |
| LVD |  | 166 | 0.97 (0.94-1.01) | 0.135 | **-** |  |
|  | | | | | |  |
| 0.0978*MVD-0.0699*LVD |  | 166 | 1.16 (0.99-1.37) | 0.071 | **-** |  |
|  | | | | | |  |

**Table S4: Univariate OS analysis**

|  | | | **Overall Survival Time (Years)** | | |
| --- | --- | --- | --- | --- | --- |
|  | | | **----------------------------------------** | | |
| **Covariate** | **Level** | **N** | **Hazard Ratio (95% CI)** | **HR P-value** | **Log-rank P-value** |
| Metastasis status | Met | 101 | 1.99 (1.37-2.88) | **<.001** | **<.001** |
|  | Non-Met | 99 | - | **-** |  |
|  | | | | | |
| Sex | Male | 130 | 1.17 (0.80-1.71) | 0.429 | 0.429 |
|  | Female | 70 | - | **-** |  |
|  | | | | | |
| Grade | WD | 32 | 1.20 (0.40-3.55) | 0.744 | 0.351 |
|  | MD | 132 | 1.71 (0.63-4.67) | 0.296 |  |
|  | PD | 26 | 1.91 (0.64-5.67) | 0.247 |  |
|  | NK | 10 | - | **-** |  |
|  | | | | | |
| Stage | I | 41 | 0.39 (0.23-0.67) | **<.001** | **0.002** |
|  | II | 31 | 0.62 (0.36-1.06) | 0.081 |  |
|  | III | 29 | 0.59 (0.35-1.00) | **0.050** |  |
|  | IV | 99 | - | **-** |  |
|  | | | | | |
| T Stage | T1 | 65 | 0.45 (0.27-0.75) | **0.002** | **0.023** |
|  | T2 | 69 | 0.66 (0.40-1.07) | 0.091 |  |
|  | T3 | 29 | 0.68 (0.38-1.20) | 0.183 |  |
|  | T4 | 37 | - | **-** |  |
|  | | | | | |
| N Stage | N0 | 99 | 0.63 (0.25-1.58) | 0.324 | **0.002** |
|  | N1 | 19 | 1.51 (0.54-4.22) | 0.430 |  |
|  | N2 | 74 | 1.22 (0.49-3.08) | 0.670 |  |
|  | N3 | 8 | - | **-** |  |
|  | | | | | |
| N Stage:binary | N0 | 99 | 0.50 (0.35-0.73) | **<.001** | **<.001** |
|  | N1-N3 | 101 | - | **-** |  |
|  | | | | | |
| Site | OP | 38 | 0.52 (0.31-0.89) | **0.018** | 0.055 |
|  | L | 61 | 0.89 (0.59-1.33) | 0.558 |  |
|  | OC | 101 | - | **-** |  |
|  | | | | | |
| Smoking | Yes | 159 | 1.24 (0.72-2.15) | 0.432 | 0.431 |
|  | No | 29 | - | **-** |  |
|  | | | | | |
| MVD:cut by ROC (High >= 40.667) | High | 65 | 1.35 (0.91-2.00) | 0.141 | 0.139 |
|  | Low | 102 | - | **-** |  |
|  | | | | | |
| MVD:cut by OS optimal (High >= 39.667) | High | 67 | 1.44 (0.97-2.13) | 0.071 | 0.070 |
|  | Low | 100 | - | **-** |  |
|  | | | | | |
| LVD:cut by ROC (High >= 3.001) | High | 132 | 0.48 (0.31-0.74) | **0.001** | **<.001** |
|  | Low | 34 | - | **-** |  |
|  | | | | | |
| LVD:cut by OS optimal (High >= 2.667) | High | 137 | 0.38 (0.24-0.60) | **<.001** | **<.001** |
|  | Low | 29 | - | **-** |  |
|  | | | | | |
| 0.0978*MVD-0.0699*LVD:cut by OS optimal (High >= 3.1247) | High | 75 | 1.48 (1.00-2.19) | **0.048** | **0.047** |
|  | Low | 91 | - | **-** |  |
|  | | | | | |
| MVD |  | 167 | 1.01 (0.99-1.02) | 0.338 | **-** |
|  | | | | | |
| LVD |  | 166 | 0.97 (0.94-1.00) | **0.045** | **-** |
|  | | | | | |
|  | | | | | |
